# Cervical and breast cancer screening participation for women with chronic conditions in France: results from a national health survey

**DOI:** 10.1186/s12885-016-2295-0

**Published:** 2016-03-31

**Authors:** Panayotis Constantinou, Rosemary Dray-Spira, Gwenn Menvielle

**Affiliations:** Sorbonne Universités, UPMC Univ Paris 06, INSERM, Institut Pierre Louis d’Epidémiologie et de Santé Publique (IPLESP UMRS 1136), F75012 Paris, France; Université Paris-Saclay, Université Paris-Sud, UVSQ, INSERM, Centre for research in Epidemiology and Population Health (CESP), Villejuif, France

**Keywords:** Cancer screening, Breast neoplasms, Uterine cervical neoplasms, Chronic disease, Comorbidity, France

## Abstract

**Background:**

Comorbidity at the time of diagnosis is an independent prognostic factor for survival among women suffering from cervical or breast cancer. Although cancer screening practices have proven their efficacy for mortality reduction, little is known about adherence to screening recommendations for women suffering from chronic conditions. We investigated the association between eleven chronic conditions and adherence to cervical and breast cancer screening recommendations in France.

**Method:**

Using data from a cross-sectional national health survey conducted in 2008, we analyzed screening participation taking into account self-reported: inflammatory systemic disease, cancer, cardiovascular disease, chronic respiratory disease, depression, diabetes, dyslipidemia, hypertension, obesity, osteoarthritis and thyroid disorders. We first computed age-standardized screening rates among women who reported each condition. We then estimated the effect of having reported each condition on adherence to screening recommendations in logistic regression models, with adjustment for sociodemographic characteristics, socioeconomic position, health behaviours, healthcare access and healthcare use. Finally, we investigated the association between chronic conditions and opportunistic versus organized breast cancer screening using multinomial logistic regression.

**Results:**

The analyses were conducted among 4226 women for cervical cancer screening and 2056 women for breast cancer screening. Most conditions studied were not associated with screening participation. Adherence to cervical cancer screening recommendations was higher for cancer survivors (OR = 1.73 [0.98–3.05]) and lower for obese women (OR = 0.73 [0.57–0.93]), when accounting for our complete range of screening determinants. Women reporting chronic respiratory disease or diabetes participated less in cervical cancer screening, except when adjusting for socioeconomic characteristics. Adherence to breast cancer screening recommendations was lower for obese women and women reporting diabetes, even after accounting for our complete range of screening determinants (OR = 0.71 [0.52–0.96] and OR = 0.55 [0.36–0.83] respectively). The lower breast cancer screening participation for obese women was more pronounced for opportunistic than for organized screening.

**Conclusion:**

We identified conditions associated with participation in cervical and breast cancer screening, even when accounting for major determinants of cancer screening. Obese women participated less in cervical cancer screening. Obese women and women with diabetes participated less in mammographic screening and organized breast cancer screening seemed to insufficiently address barriers to participation.

**Electronic supplementary material:**

The online version of this article (doi:10.1186/s12885-016-2295-0) contains supplementary material, which is available to authorized users.

## Background

Chronic disease morbidity is an issue of increasing importance for cancer research [1]. While chronic conditions are already the leading cause of death globally and their burden is expected to increase [2], it has now been shown that all-cause mortality as well as cancer-specific mortality is higher for newly diagnosed cancer patients suffering from chronic conditions, even when stage at diagnosis or treatment are taken into account [3, 4]. More specifically, comorbidity at the time of diagnosis is an independent prognostic factor for survival among both cervical cancer [5, 6] and breast cancer patients [7, 8]. A recent study showed that the presence of one chronic condition was equivalent to one tumor stage shift in terms of breast cancer survival decrease [9].

Among available tools for cancer control, cervical smears have proved their efficacy to reduce cervical cancer incidence and mortality [10, 11]. For breast cancer, although the portion of mortality reduction attributable to screening has been subject to controversy [12, 13], recent studies have found a 10 to 20 % reduction in breast cancer mortality among women who underwent mammographic screening [14–16]. In France, cervical cancer screening is recommended every three years for women aged 25 to 65 years and is based on individual cervical smear use (opportunistic screening). A nationwide organized breast cancer screening has been implemented in 2004 and women aged 50 to 74 years are individually invited to attend mammography screening, free of charge, every two years. This organized program exists alongside opportunistic screening, since individual prescriptions of mammograms are reimbursed.

Yet, inconsistent results have been reported regarding adherence to recommended screening procedures among patients suffering from chronic diseases [17]. Some conditions are generally associated with higher cancer screening rates (e.g. cancer survivors [18]), others with lower cancer screening rates (e.g. diabetes [19]) and contradictory results are reported for conditions such as rheumatoid arthritis [20, 21]. When the overall effect of chronic morbidity on cervical and breast cancer screening is studied using summary measures, increased comorbidity is associated with decreased screening in clinic-based studies [21] and with increased screening in population-based studies [22]. In addition, these studies did not systematically investigate the factors explaining the association between the presence of chronic diseases and cancer screening participation. Evidence on screening determinants is now extensive [23] and a large range of variables are associated with smear use or mammography, including demographic and socioeconomic characteristics, health behaviours and healthcare related variables [24, 25]. There is also evidence that fewer factors are associated with screening participation when organized programs exist. In particular, women with lower socioeconomic positions are more likely to attend screening through organized programs than through opportunistic screening [26–28].

In this context, our primary objective was to identify chronic conditions associated with adherence to cervical and breast cancer screening recommendations in France and to investigate whether these associations were modified by several major cancer screening determinants. Our secondary objective was to explore whether the associations between chronic conditions and breast cancer screening participation were specific to opportunistic or organized screening.

## Methods

### Data source

Our study was based on data from the 2008 wave of the Healthcare and Health Insurance Survey (*Enquête Santé et Protection Sociale*), a national health survey conducted by the Institute for Research and Information on Health Economics. Information was collected among a random sample of non-institutionalized health-insured people living in mainland France and from all the members of their households. The overall sample included 22,273 individuals spread over 8,257 households. All individuals were interviewed to collect information on sociodemographic characteristics and received a questionnaire for health-related questions and screening behavior. Overall response rate to this self-reported health questionnaire was 72 % [29].

### Outcome

**Fig. 1:**
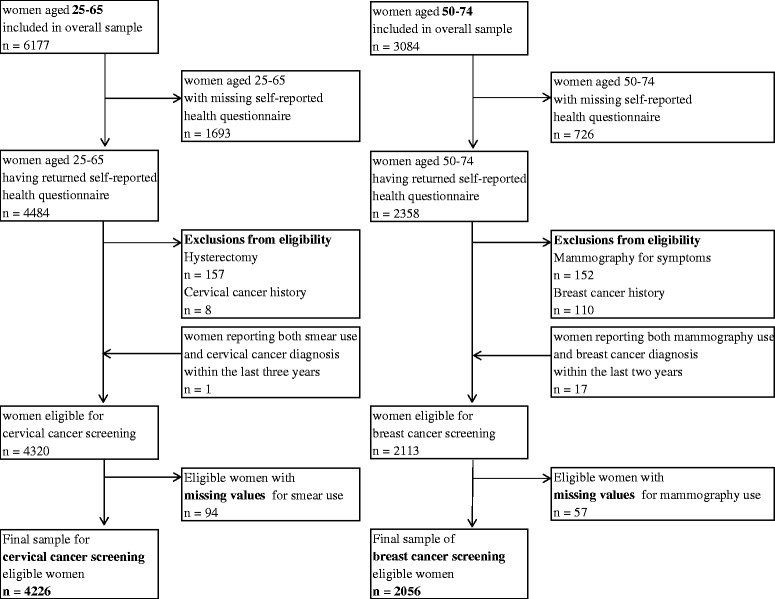
Flowcharts describing the cervical (left panel) and breast cancer (right panel) screening sample selection

The two outcomes were adherence to the French Health authority’s cervical and breast cancer screening recommendations: having undergone a cervical smear within the last three years for women aged 25 to 65 years and having undergone a mammography within the last two years for women aged 50 to 74 years. The reason for undergoing mammography was available, which allowed us to distinguish opportunistic from organized screening participation. Official exclusion criteria were applied. Women who reported both cancer diagnosis and last screening use within the recommended interval were not excluded, as cancer could have been diagnosed during the last screening, and thus does not constitute an exclusion criterion. The selection process for the studied samples is presented in Fig. 1.

### Chronic conditions

Morbidity at the time of the survey was self-reported from among an extensive checklist of more than 50 conditions, with the possibility of free text declarations. For each reported condition, the respondents indicated if they had been treated within the last 12 months. For each respondent, the list of reported chronic conditions was retrospectively validated by a physician, as part of the Healthcare and Health Insurance Survey study, using answers to questions including past 24 hours’ medication consumption, history of surgery or prosthetics, reason for last medical appointment or long-term illness fee exemption (corresponding to the full reimbursement of medical fees for a specific condition). For the purpose of this analysis, we reviewed all the conditions reported to define the eleven most common and mutually exclusive chronic conditions: inflammatory systemic disease (arthritis or vascularitis or inflammatory bowel disease), cancer (other than: cervical cancer, for cervical cancer screening sample and breast cancer, for breast cancer screening sample), cardiovascular diseases, chronic respiratory diseases, depression, diabetes, dyslipidemia, hypertension, obesity, osteoarthritis and thyroid disorders. For depression, dyslipidemia and hypertension, we restricted the selection to women who reported having been treated within the last 12 months, due to the poor specificity of these self-reported conditions. Obesity was defined using body mass index (BMI), calculated using self-reported weight and height (obesity if BMI > =30 kg/m^2^).

### Covariates

To investigate whether the association between chronic conditions and screening participation was modified by the major screening determinants, we classified adjustment variables into five acknowledged categories of determinants [23, 25]. We selected the variables significantly associated in univariate analysis with screening participation and with the majority of studied conditions. We then assessed pairwise correlation between variables within each category of determinants and multicollinearity among all variables to define the final list of covariates. The following groups were defined: *sociodemographic characteristics*: age (categorized for breast cancer in 5-year groups and for cervical cancer as follows: 25–39, 40–49 and then 5-year groups), household composition (single adult without children/couple without children/single adult with children/couple with children); *socioeconomic position*: highest educational level attained (primary education or less/did not graduate high school/graduated high school/higher than high school), housing tenure (renter/owner with mortgage/outright owner), employment status (inactive/employed/unemployed/retired); *health behaviours*: smoking (never smokers/current smokers/ex-smokers); *healthcare access*: complementary health insurance status (none/private/free coverage for low income individuals), long-term illness fee exemption (yes/no); *healthcare use*: physicians consulted within the last 12 months (at least one gynecologist/other physician(s)/none)

### Statistical analyses

All analyses were conducted for cervical and breast cancer screening separately. We first computed age-standardized screening rates among the subgroups of women suffering from each chronic condition of interest, with direct standardization, using the age distribution of the entire eligible population as standard.

We then compared screening participation between women with each chronic condition of interest versus women without the condition, using logistic regression modeling. For all models, adherence to screening recommendations was the dependent variable and chronic conditions were specified as dichotomous explanatory variables. All models were systematically adjusted for age. Models were also adjusted for our five categories of determinants: sociodemographic characteristics, socioeconomic position, health behaviours, healthcare access and healthcare use, first separately and then simultaneously in a fully-adjusted model.

Additional analyses were conducted to disentangle the effect of the chronic condition of interest from that of other concomitant conditions: (i) for each condition, we additionally adjusted our fully-adjusted model for the number of conditions reported; (ii) we studied the association between screening participation and each condition when coded as a categorical variable (condition reported alone or with 1, 2 or 3 or more other conditions among the 11 conditions studied).

Finally, the association between breast cancer screening and the presence of chronic conditions was investigated by type of screening (organized versus opportunistic) using multinomial logistic regression.

For the covariates, missing values were rare (<4 %) except for smoking (10.9 % in the cervical cancer screening sample and 19.0 % in the breast cancer screening sample) and were treated as a separate category in the analyses. For the chronic condition variables, there were no missing values except for obesity (4 %). These women were considered as a separate category. Sensitivity analyses showed similar results when classifying missing values as obese or non-obese. Available sampling weights to account for the survey’s sampling design and overall non-response were applied and our estimates can be extrapolated to the entire non-institutionalized French mainland population living in households.

All statistical analyses were performed using Stata 11 software in survey mode (StataCorp. 2009. *Stata Statistical Software: Release 11*. College Station, TX: StataCorp LP).

### Ethics

The Healthcare and Health Insurance Survey waves conducted biennially by the Institute for Research and Information on Health Economics (IRDES) are approved by the national administrative authority on data protection (*CNIL, Commission Nationale de l’Informatique et des Libertés*, authorization n°1147702-V2). Databases are available upon request and research collaboration with the IRDES. Written informed consent was not required for this study as all data were anonymized.

## Results

### Study population

The cervical cancer screening sample included 4,226 women aged 25–65 years and the breast cancer screening sample included 2,056 women aged 50–74 years (Fig. 1). Prevalence of the studied conditions among cervical and breast cancer screening eligible women are presented in Tables 1 and 2, respectively. The most prevalent reported conditions were osteoarthritis (reported by 16.0 % of women in the cervical cancer screening sample and 35.8 % of women in the breast cancer screening sample), obesity (12.1 % and 15.3 % respectively) and hypertension (8.1 % and 18.8 % respectively). The distribution of covariates among eligible women is available in Additional file 1: Table S1.

**Table 1:** Prevalence of and cervical cancer screening rate for the studied conditions

	Sample size		Age		
	N	%^a^	(mean,σ)	N	rate^b^ [95 % CI]
All women	4226	100	44,6 (11,0)	3201	75,8 [74,5-77,2]
Inflammatory systemic disease^c^	98	2,4	48,9 (10,8)	66	72,5 [63,8-81,2]
Cancer^d^	122	3,0	52,3 (9,2)	98	80,1 [69,3-91,0]
Cardiovascular disease	87	2,1	51,7 (9,5)	59	74,0 [64,7-83,2]
Chronic Respiratory disease	294	7,2	45,4 (11,4)	199	67,7 [62,3-73,2]
Depression^e^	177	4,7	46,7 (10,3)	137	76,3 [69,3-83,4]
Diabetes	122	2,9	54,1 (8,0)	67	63,6 [51,3-76,0]
Dyslipidemia^e^	167	4,1	55,0 (7,4)	117	74,7 [63,9-85,5]
Hypertension^e^	331	8,1	53,7 (8,1)	228	74,5 [68,1-80,9]
Obesity	518	12,1	46,5 (11,2)	326	65,0 [60,7-69,3]
Osteoarthritis	653	16,0	52,4 (8,8)	465	74,1 [69,8-78,3]
Thyroid disorders	323	7,6	48,3 (9,8)	248	77,6 [72,7-82,4]

^a^weighted percentages (to account for the survey’s sampling design and overall non-response)

^b^age-standardized weighted rates (the age distribution of “all women” was used as standard for each screening)

^c^arthritis or vascularitis or inflammatory bowel disease

^d^other than cervical cancer

^e^treated within the last 12 months

**Table 2:** Prevalence of and breast cancer screening rate for the studied conditions

	Sample size		Age	Overall screening		Organized screening		Opportunistic screening	
	N	%^a^	(mean,σ)	N	rate^b^ [95 % CI]	N	rate^b^ [95 % CI]	N	rate^b^ [95 % CI]
All women	2056	100	60,2 (7,0)	1533	74,9 [73.0-76.9]	1091	54,4 [52,2-56,6]	374	17,2 [15.6-18.8]
Inflammatory systemic disease^c^	84	4,3	62,7 (7,2)	67	79,3 [70,2-88,4]	50	58,3 [47,0-69,5]	13	16,6 [1, 1-8, 8-25]
Cancer^d^	67	3,4	63,1 (7,0)	48	76,0 [65,8-86,2]	33	51,4 [38,5-64,3]	11	20,1 [9,1-31,0]
Cardiovascular disease	96	4,9	64,0 (7,4)	69	69,7 [59,8-79,7]	57	56,4 [46,7-66,1]	9	10,9 [3,1-18,7]
Chronic Respiratory disease	173	8,8	61,5 (7,0)	124	69,0 [61,7-76,2]	106	57,8 [50,2-65,4]	14	9,3 [4,3-14,2]
Depression^e^	85	4,7	59,1 (6,6)	58	66,1 [55,3-77,0]	36	46,5 [35,4-57,5]	17	14,6 [8,3-21,0]
Diabetes	157	7,6	62,7 (7,4)	91	59,7 [51,4-67,9]	64	42,1 [33,8-50,4]	20	14,1 [8,1-20,0]
Dyslipidemia^e^	234	11,8	63,2 (6,9)	179	77,9 [72,0-83,8]	143	62,7 [55,9-69,5]	33	14,1 [9,2-18,9]
Hypertension^e^	369	18,8	62,0 (7,1)	279	76,2 [71,7-80,9]	216	59,2 [54,0-64,5]	55	15,2 [11,4-19,0]
Obesity	319	15,3	61,0 (6,9)	212	67,2 [61,8-72,6]	167	53,9 [48,3-59,6]	38	11,3 [7,8-14,8]
Osteoarthritis	707	35,8	62,4 (7,1)	539	75,9 [72,4-79,3]	410	57,3 [53,4-61,1]	108	15,8 [12,9-18,7]
Thyroid disorders	253	12,5	62,0 (7,2)	194	76,0 [70,4-81,7]	140	53,6 [47,2-60,1]	45	19,3 [14,1-24,4]

^a^weighted percentages (to account for the survey’s sampling design and overall non-response)

^b^age-standardized weighted rates (age distribution of “all women” was used as standard for each screening)

^c^arthritis or vascularitis or inflammatory bowel disease

^d^other than breast cancer

^e^treated within the last 12 months

Note: some respondents reported mammography use within the last two years without precision on screening mode. Therefore, the number of women who participated in organized and opportunistic breast cancer screening may not always add up to the overall number of women who participated in breast cancer screening

### Cervical cancer screening (Tables 1 and 3)

**Table 3:** Odds ratios^a^ of cervical cancer screening participation for eleven chronic conditions

	Models adjusted for						
	Age	Age and sociodemographic characteristics^b^	Age and socioeconomic position^c^	Age and health behaviours^d^	Age and healthcare access^e^	Age and healthcare use^f^	Fully-adjusted^g^
	OR [95 % CI]						
							OR [95 % CI]
					OR [95 % CI]	OR [95 % CI]	
			OR [95 % CI]	OR [95 % CI]			
		OR [95 % CI]					
Inflammatory systemic disease^h^	0.82 [0.53-1.29]	0,78 [0,49-1,25]	0,82 [0,50-1,35]	0,81 [0,51-1,27]	0,83 [0,52-1,33]	0.88 [0.55-1.42]	0,82 [0,48-1,39]
Cancer^i^	1.53 [0.94-2.51]	1,54 [0,95-2,51]	1,76 [1,04-2,98]	1,63 [1,00-2,68]	2,00 [1,20-3,34]	1.38 [0.82-2.32]	1,73 [0,98-3,05]
Cardiovascular diseases	0.77 [0.47-1.25]	0,81 [0,50-1,34]	0,93 [0,57-1,52]	0,81 [0,49-1,34]	0,91 [0,54-1,51]	0.84 [0.50-1.41]	1,07 [0,61-1,86]
Chronic respiratory diseases	0.65 [0.49-0.85]	0,71 [0,54-0,93]	0,79 [0,60-1,05]	0,67 [0,51-0,87]	0,72 [0,54-0,96]	0.64 [0.48-0.85]	0,82 [0,60-1,13]
Depression^j^	1.11 [0.75-1.65]	0,27 [0,85-1,89]	1,35 [0,90-2,03]	1,16 [0,79-1,72]	1,27 [0,84-1,91]	1.05 [0.68-1.62]	1,33 [0,84-2,09]
Diabetes	0.49 [0.33-0.72]	0,53 [0,35-0,78]	0,72 [0,48-1,09]	0,50 [0,34-0,74]	0,56 [0,36-0,85]	0.53 [0.34-0.83]	0,71 [0,44-1,15]
Dyslipidemia^j^	0.98 [0.68-1.43]	1,01 [0,70-1,47]	1,08 [0,74-1,57]	0,95 [0,66-1,39]	1,04 [0,71-1,51]	0.73 [0.49-1.11]	0,79 [0,53-1,19]
Hypertension^j^	0.89 [0.68-1.16]	0,91 [0,69-1,20]	0,95 [0,72-1,26]	0,87 [0,66-1,14]	0,92 [0,70-1,21]	0.86 [0.64-1.15]	0,92 [0,68-1,25]
Obesity	0.52 [0.43-0.65]	0,56 [0,45-0,69]	0,66 [0,53-0,83]	0,52 [0,42-0,65]	0,58 [0,46-0,72]	0.61 [0.48-0.76]	0,73 [0,57-0,93]
Osteoarthritis	0.91 [0.74-1.12]	0,95 [0,77-1,17]	1,06 [0,86-1,32]	0,90 [0,73-1,10]	0,97 [0,78-1,19]	0.85 [0.68-1.06]	0,96 [0,76-1,21]
Thyroid disorders	1.12 [0.85-1.49]	1,12 [0,85-1,48]	1,14 [0,85-1,53]	1,11 [0,84-1,48]	1,12 [0,84-1,48]	1.03 [0.76-1.39]	1,04 [0,76-1,42]

*OR* odds ratio

95 % CI: 95 % confidence interval

^a^the reference category is women who have not reported the condition of interest

^b^adjusted for age and household composition

^c^adjusted for age and highest educational level attained, housing tenure, employment status

^d^adjusted for age and smoking

^e^adjusted for age and complementary health insurance status, long-term illness fee exemption

^f^adjusted for age and physician consulted within the last 12 months

^g^adjusted for all covariates

^h^arthritis or vascularitis or inflammatory bowel disease

^i^other than cervical cancer

^j^treated within the last 12 months

The overall cervical cancer screening rate was 75.8 % [95 % confidence interval 74,5-77,2]. When compared with the whole population, age-standardized screening rates were lower among women reporting chronic respiratory diseases (67.7 % [62.3–73.2]) and among obese women (65.0 % [60.7–69.3]). Although not statistically significant, the lowest screening rate was observed among women reporting diabetes (63.6 % [51.3–76.0]) (Table 1). For each chronic condition of interest, we present in Table 3 the odds ratios (OR) of screening participation for women having versus not having reported this condition. Women with a history of cancer were more likely to adhere to screening recommendations. The association was nevertheless markedly reduced when accounting for healthcare use (OR = 1.38 [0.82–2.32]). Lower adherence to screening recommendations was found for women reporting chronic respiratory diseases, diabetes or obesity. Socioeconomic factors accounted for this lower participation for chronic respiratory diseases (OR = 0.79 [0.60–1.05]) and diabetes (OR = 0.72 [0.48–1.09]). For obese women however, statistically significant lower screening participation was still observed in the fully adjusted model (OR = 0.73 [0.57–0.93]). Our results were robust to further adjustment for the number of conditions reported among the 11 studied (results not shown). The association was even strengthened for women reporting obesity or cancer without any additional comorbidity (OR = 0.56 [0.39–0.79] and OR = 3.17 [1.24–8.10] respectively, in the fully-adjusted model).

### Breast cancer screening (Tables 2, 4 and 5)

**Table 4:** Odds ratios^a^ of breast cancer screening participation for eleven chronic conditions

	Models adjusted for						
	Age	Age and sociodemographic characteristics^b^	Age and socioeconomic position^c^	Age and health behaviours^d^	Age and healthcare access^e^	Age and healthcare use^f^	Fully-adjusted^g^
	OR [95 % CI]						
							OR [95 % CI]
					OR [95 % CI]	OR [95 % CI]	
			OR [95 % CI]	OR [95 % CI]			
		OR [95 % CI]					
Inflammatory systemic disease^h^	1.29 [0.73-2.28]	1,27 [0,72-2,24]	1,28 [0,73-2,27]	1,24 [0,70-2,19]	1,48 [0,83-2,65]	1.46 [0.79-2.68]	1,51 [0,82-2,78]
Cancer^i^	0.91 [0.51-1.60]	0,94 [0,53-1,66]	0,97 [0,54-1,74]	0,92 [0,52-1,63]	1,17 [0,64-2,16]	0.85 [0.48-1.51]	1,10 [0,58-2,06]
Cardiovascular diseases	0.93 [0.57-1.51]	0,93 [0,57-1,52]	1,08 [0,65-1,78]	0,97 [0,60-1,58]	1,19 [0,72-1,97]	0.90 [0.55-1.47]	1,22 [0,72-2,05]
Chronic respiratory diseases	0.79 [0.54-1.14]	0,81 [0,56-1,17]	0,94 [0,65-1,36]	0,81 [0,56-1,17]	0,91 [0,62-1,32]	0.79 [0.54-1.15]	0,96 [0,64-1,42]
Depression^j^	0.73 [0.44-1.20]	0,77 [0,46-1,29]	0,88 [0,54-1,44]	0,74 [0,45-1,22]	0,89 [0,53-1,48]	0.67 [0.39-1.15]	0,84 [0,48-1,45]
Diabetes	0.46 [0.32-0.65]	0,48 [0,33-0,68]	0,52 [0,36-0,74]	0,44 [0,31-0,63]	0,53 [0,35-0,79]	0.48 [0.33-0.70]	0,55 [0,36-0,83]
Dyslipidemia^j^	1.17 [0.83-1.64]	1,20 [0,86-1,69]	1,25 [0,89-1,77]	1,16 [0,82-1,64]	1,21 [0,86-1,71]	1.05 [0.73-1.49]	1,13 [0,78-1,62]
Hypertension^j^	1.06 [0.80-1.39]	1,07 [0,81-1,41]	1,10 [0,83-1,46]	1,02 [0,77-1,36]	1,09 [0,82-1,45]	1.01 [0.76-1.35]	1,03 [0,76-1,39]
Obesity	0.61 [0.46-0.80]	0,62 [0,47-0,81]	0,69 [0,52-0,92]	0,59 [0,45-0,78]	0,65 [0,49-0,86]	0.64 [0.48-0.86]	0,71 [0,52-0,96]
Osteoarthritis	1.13 [0.90-1.42]	1,15 [0,92-1,45]	1,21 [0,96-1,54]	1,12 [0,89-1,41]	1,17 [0,93-1,47]	1.09 [0.86-1.38]	1,16 [0,91-1,48]
Thyroid disorders	1.13 [0.81-1.57]	1,11 [0,80-1,55]	1,04 [0,75-1,45]	1,14 [0,82-1,58]	1,19 [0,85-1,67]	0.99 [0.71-1.40]	1,00 [0,70-1,41]

*OR* odds ratio

95 % CI: 95 % confidence interval

^a^the reference category is women who have not reported the condition of interest

^b^adjusted for age and household composition

^c^adjusted for age and highest educational level attained, housing tenure, employment status

^d^adjusted for age and smoking

^e^adjusted for age and complementary health insurance status, long-term illness fee exemption

^f^adjusted for age and physician consulted within the last 12 months

^g^adjusted for all covariates

^h^arthritis or vascularitis or inflammatory bowel disease

^i^other than breast cancer

^j^treated within the last 12 months

**Table 5:** Odds ratios^a^ of participation in organized and opportunistic breast cancer screening for selected chronic conditions

	No screening	Organized screening	Opportunistic screening	Wald test^b^ (p)
		OR [95 % CI]	OR [95 % CI]	
Chronic Respiratory disease				
Age-adjusted model	1	0,92 [0,63-1,34]	0,42 [0,22-0,81]	0,01
Age and socioeconomic position-adjusted model^c^	1	1,08 [0,74-1,57]	0,53 [0,27-1,01]	0,02
Fully-adjusted model^d^	1	1,11 [0,74-1,66]	0,54 [0,27-1,11]	0,03
Diabetes				
Age-adjusted model	1	0,45 [0,30-0,65]	0,47 [0,27-0,81]	0,85
Age and socioeconomic position-adjusted model^c^	1	0,49 [0,34-0,73]	0,60 [0,34-1,05]	0,51
Fully-adjusted model^d^	1	0,50 [0,32-0,79]	0,71 [0,37-1,36]	0,25
Obesity				
Age-adjusted model	1	0,68 [0,51-0,91]	0,42 [0,27-0,63]	0,01
Age and socioeconomic position-adjusted model^c^	1	0,77 [0,57-1,04]	0,51 [0,33-0,78]	0,04
Fully-adjusted model^d^	1	0,78 [0,57-1,08]	0,54 [0,34-0,86]	0,08

^a^ORs refer to women having reported the condition of interest when compared to women who have not reported this condition

^b^tests the significance of the difference between the ORs for organized and opportunistic screening (regardless of the significance of each OR)

^c^adjusted for age and highest educational level attained, housing tenure, employment status

^d^adjusted for all covariates

The overall breast cancer screening rate was 74.9 % [73.0–76.9]. When compared with the whole population, age-standardized screening rates were lower among women reporting diabetes (59.7 % [51.4–67.6]) and among obese women (67.2 % [61.8–72.6]) (Table 2). Significantly lower adherence to screening recommendations was observed for women reporting diabetes or obese women in all models (OR = 0.55 [0.36–0.83] for diabetes, OR = 0.71 [0.52–0.96] for obesity, in the fully-adjusted model). Our results were robust to further adjustment for the number of conditions reported among the 11 studied (results not shown). Associations were strengthened for women reporting diabetes or obesity without any additional comorbidity (OR = 0.33 [0.13–0.82] and OR = 0.41 [0.21–0.78] respectively in the fully-adjusted model). As diabetes and obesity are frequently associated, we further investigated the association between breast cancer screening participation and combined or independent exposure to diabetes and obesity. When compared to women reporting neither diabetes nor obesity, lower screening rates were observed for women reporting both conditions (OR = 0.41 [0.23–0.73] in the fully-adjusted model) or diabetes alone (OR = 0.54 [0.31–0.92]) whereas the association was not statistically significant for women reporting obesity alone (OR = 0.74 [0.53–1.04]).

Table 5 presents the association between participation in organized and opportunistic breast cancer screening and chronic conditions. We only present results adjusted for age, for age and socioeconomic position and for all screening determinants and for conditions with significant estimates. Women reporting chronic respiratory diseases participated less in opportunistic screening only. Lower screening rates were found for both types of screening among women reporting diabetes and among obese women. This lower participation was nevertheless more pronounced for opportunistic screening for obese women (OR = 0.54 [0.34–0.86] vs OR = 0.78 [0.57–1.08] in the fully-adjusted model).

## Discussion

Adherence to cervical cancer screening recommendations was higher for cancer survivors and lower for obese women when compared to women who did not report these conditions. Lower participation in cervical cancer screening was also observed for women reporting chronic respiratory diseases or diabetes, except when adjusting for socioeconomic characteristics. Adherence to breast cancer screening recommendations was lower for obese women and women reporting diabetes, even after accounting for our complete range of screening determinants. The lower breast cancer screening participation for obese women was more pronounced for opportunistic than for organized screening.

### Findings in relation to other studies and interpretation

Only few studies investigated the association between a large range of chronic conditions and cancer screening as we did and they also suggested that most conditions were not associated with screening participation [21, 22]. Contrasted findings were nevertheless observed for rheumatoid arthritis [20, 21] and respiratory diseases, with differences between asthma and COPD [21]. Consistent with our findings, most studies [18], although not all [22], reported that adherence to recommended cancer screening practices was higher among cancer survivors. Lower cervical and breast cancer screening use has also been repeatedly reported among women with diabetes, both in clinic-based and population-based studies [19, 21, 30], as well as among obese women [31, 32]. However, the available literature reported that hypertension was associated with an increased cervical cancer screening [21, 22], an association that we did not find.

Cancer is among the rare chronic conditions associated with higher cervical smear or mammography use. However, it is unclear whether this association remains when breast (for cervical smear) or cervical (for mammography) cancer survivors are excluded [18]. We observed that higher cervical cancer screening participation was restricted to breast cancer survivors (results not shown) and was largely accounted for by healthcare use. We therefore hypothesize that a history of breast cancer is likely to induce a more frequent gynecological follow-up that in turn largely accounts for the higher cervical cancer screening participation for breast cancer survivors.

Obesity was significantly associated with lower participation in cervical and breast cancer screening in our study, even when accounting for a large range of determinants. Qualitative research underlines that obese women face both the usual patient-related barriers to screening, such as fear or embarrassment, and specific weight-related barriers, such as inadequate equipment or negative interaction with physicians [33]. We compared obese to non-obese women. However, consistent with the literature [34], we found an inverse gradient between BMI and cervical or breast cancer screening uptake (results not shown). The lower cancer screening uptake that we observed for obese women would then have been more pronounced had we compared obese to normal weight women.

Both obesity and diabetes are associated with a higher risk of postmenopausal breast cancer. Numerous studies have reported a gradient between BMI and postmenopausal breast cancer risk: compared with normal weight women, cancer risk is significantly higher for overweight women and continues to rise for obese women [35]. For diabetes, the association with breast cancer is more modest and the carcinogenesis mechanism is less clear than the exposure to elevated circulating estrogen levels in obese women. However, a recent meta-analysis concluded in a significant association between type II diabetes and postmenopausal breast cancer, persistent when adjusting for BMI [36]. Obese women or women with diabetes are therefore at the same time less covered by mammographic prevention and more exposed to postmenopausal breast cancer risk.

Women suffering from chronic conditions are more likely to have a regular medical follow-up. This may be particularly true for conditions with standardized follow-up procedures such as diabetes. The possible role of medical follow-up in adherence to cancer screening recommendations, however, is not clear. It has been suggested that physicians concentrated on specific chronic disease management at the expense of other preventive care practices, including cancer screening, both among diabetic women [37] or obese people [38]. On the other hand, there is evidence that cancer screening rates increase with increasing number of chronic conditions [39], suggesting that competing demand is not a sufficient explanation for lower screening participation among women with diabetes or obesity. In addition, a more frequent medical follow-up has been associated with higher cancer screening rates among individuals with diabetes [19, 37] and with more physician recommendations for cervical smear among obese women [34]. Finally, although similar findings are observed for obese and diabetic women, obesity does not require a medical diagnosis and many obese women are not treated for obesity until they develop another chronic condition. In our study, healthcare use did not explain the lower screening participation for women with diabetes or obese women. The relevant factor may not be the frequency of medical follow-up but the quality of care. Indeed, there is evidence that among women with diabetes, the adherence to screening recommendations is associated to the quality of diabetes-related processes of care [40]. Our measure of medical follow-up, however, did not allow us to study the quality of processes of care.

Socioeconomic status accounted for the lower cervical cancer screening participation for women reporting chronic respiratory diseases or diabetes in our study. This effect was expected, as low socioeconomic status is associated with both lower screening rates [41] and most of the conditions investigated, in particular diabetes [42]. Studies also report that organized compared to opportunistic screening led to decreased socioeconomic inequalities in screening participation [26, 27]. This is likely to explain why socioeconomic characteristics had a larger effect on cervical cancer screening participation, where no nationwide organized screening program was available, than on breast cancer screening participation. Also, although estimates of the screening mode comparisons for breast cancer should be considered with caution because of small sample sizes, we found a similar pattern between cervical cancer screening and opportunistic breast cancer screening, with socioeconomic position accounting for the lower participation for women reporting chronic respiratory disease or diabetes.

Although a recent evaluation suggests that exclusive organized screening would be more efficient than the actual coexistence of both screening modes in France [43], lower organized breast cancer screening participation was observed for women reporting diabetes. Our findings therefore suggest that other factors than those investigated in our study still constitute barriers to screening, which organized screening may not have yet been able to address.

### Strengths and limitations

We used data from a large national survey, taking advantage of the survey’s overall sample size to study a substantial number of chronic conditions and explanatory variables. Our objective was to analyze the association between common chronic conditions and recommended cancer screening use but due to too small sample sizes, we could not restrict our analyses to women suffering from one single condition. In order to investigate the independent effect of each condition we conducted several additional analyses to test the robustness of our results: we accounted for the number of chronic conditions reported, as some studies found that the association between cancer screening participation and chronic conditions disappeared with this adjustment [21]; we studied the association with screening participation when the conditions were reported without additional disease; and we investigated the combined and independent effect of diabetes and obesity. The stability of our results across all analyses strongly supports an independent effect of the identified chronic conditions on screening participation.

The quality of our data should be discussed. As 25 % of women aged 25–74 included in the survey did not return the health questionnaire and therefore did not provide information on chronic conditions and screening participation, selection bias is an issue. However, a proxy for chronic condition ascertainment, the report of a long-term illness fee exemption, as well as most screening determinants (except smoking and visits to physicians within the last year) were available for all respondents. We assessed the magnitude of the selection bias by comparing the distribution of these covariates between women who did and did not return the health questionnaire. The distribution of the proxy for chronic condition ascertainment did not differ between these two groups. Regarding the screening determinants, response rate was lower among women under the age of 40, living in couple with children, inactive, without complementary health insurance and higher among women over the age of 60, living alone without children, retired, with free insurance for low income. However, women with higher (lower) response rate were not systematically those with higher (lower) screening participation (Additional file 1: Table S1). We therefore believe that the selection bias is not likely to account for our results.

Screening participation and morbidity data were self-reported. Although self-reported cervical and breast cancer screening participation is thought to overestimate actual use, population-based surveys with questionnaires could be considered the most valid and the accuracy of self-reporting does not seem to be associated with socioeconomic factors [44]. We lack data however on the accuracy of self-reported screening participation according to the presence of chronic conditions. Finally, although chronic disease ascertainment was validated by physicians and in spite of the great attention given to the definition of the studied conditions, data on chronic diseases may still suffer from bias. First, because of the cross-sectional design of the survey, the chronic condition may not always have been present at the time of screening and prevalence could be overestimated. Second, the validity of chronic disease ascertainment with population-based data depends on the conditions studied. The strongest agreement with administrative data is observed for diabetes or hypertension but prevalence of conditions such as arthritis or heart disease may be underestimated in self-reported population-based studies, with a tendency to identify less healthy people [45, 46]. It has also been shown that BMI was underestimated when self-reported, especially among obese or elderly people [47, 48].

Overall, we believe that our results, especially for diabetes and obesity, are not likely to be explained by self-reporting bias. Finally, although consistent with the literature, we cannot rule out that for the majority of the conditions studied, the lack of association with cervical and breast cancer screening participation may be explained by less accurate self-reporting, especially for conditions with intermittent and non-specific symptoms (e.g. chronic respiratory conditions) or non-life-threatening conditions (e.g. arthritis) [45, 46].

## Conclusion

Cancer screening is only one among the available tools for cancer control but it has proven its efficacy for cervical and breast cancer mortality reduction. We identified conditions associated with cervical and breast cancer screening participation and investigated the determinants explaining these associations. Obese women participated less in cervical cancer screening use and, although at higher risk of postmenopausal breast cancer, obese women and women reporting diabetes are less likely to follow mammographic screening recommendations. Noteworthy, organized breast cancer screening seems to insufficiently address barriers to screening among these exposed populations. Further investigation is needed to better understand cancer screening determinants among chronically ill women and to design interventions that efficiently increase screening coverage in these groups.

## Additional file

Additional file 1: Table S1. Characteristics of women eligible for cervical and breast cancer screening. (DOCX 30 kb)
