# Supplementary material for: Cervical and breast cancer screening participation for women with chronic conditions in France: results from a national health survey
Source: BMC Cancer. 2016 Mar 31;16:255. doi: 10.1186/s12885-016-2295-0 (PMC4815180; doi:10.1186/s12885-016-2295-0)
Supplement: Additional file 1: Table S1. — Characteristics of women eligible for cervical and breast cancer screening. (DOCX 30 kb) [file 12885_2016_2295_MOESM1_ESM.docx]

| ***Table S1. Characteristics of women eligible for cervical and breast cancer screening*** | | | | | | | |  |
| --- | --- | --- | --- | --- | --- | --- | --- | --- |
|  | **Cervical cancer screening** | | | | **Breast cancer screening** | | | |
|  | **N^a^** | **%^b^** | **Rate^c^** | **p^d^** | **N^a^** | **%^b^** | **Rate^c^** | **p^d^** |
| **full sample of eligible women** | **4226** | **100** | **75,8** |  | **2056** | **100** | **74,9** |  |
|  |  |  |  |  |  |  |  |  |
| **SOCIODEMOGRAPHIC CHARACTERISTICS** | |  |  |  |  |  |  |  |
| **age cervix breast** |  |  |  | p<0,001 |  |  |  | p=0,018 |
| 25-39 50-54 | 1478 | 35,7 | 82,0 |  | 570 | 25,5 | 73,3 |  |
| 40-49 55-59 | 1199 | 25,6 | 75,8 |  | 447 | 20,5 | 76,8 |  |
| 50-54 60-64 | 602 | 14,2 | 73,7 |  | 472 | 24,1 | 78,5 |  |
| 55-59 65-69 | 452 | 11,1 | 70,6 |  | 261 | 13,4 | 76,8 |  |
| 60-65 70-74 | 495 | 13,4 | 66,0 |  | 306 | 16,5 | 68,4 |  |
|  |  |  |  |  |  |  |  |  |
| **household composition** |  |  |  | p<0,001 |  |  |  | p<0,001 |
| single adult without children | 348 | 15,0 | 68,4 |  | 296 | 23,9 | 71,6 |  |
| couple without children | 1054 | 25,4 | 76,9 |  | 1101 | 49,5 | 78,6 |  |
| single adult with children | 423 | 8,8 | 61,7 |  | 135 | 5,7 | 64,4 |  |
| couple with children | 2259 | 48,0 | 81,0 |  | 448 | 18,1 | 75,4 |  |
| missing | 142 | 2,8 | 60,0 |  | 76 | 2,9 | 57,4 |  |
|  |  |  |  |  |  |  |  |  |
| **SOCIOECONOMIC POSITION** |  |  |  |  |  |  |  |  |
| **highest educational level attained** |  |  |  | p<0,001 |  |  |  | p<0,001 |
| primary education or less | 692 | 16,2 | 58,8 |  | 726 | 35,8 | 68,3 |  |
| did not graduate high school | 1445 | 33,1 | 73,7 |  | 672 | 32,3 | 78,2 |  |
| graduated high school | 657 | 15,7 | 80,3 |  | 254 | 12,7 | 76,9 |  |
| higher than high school | 1336 | 32,9 | 84,7 |  | 352 | 17,0 | 80,3 |  |
| missing | 96 | 2,1 | 68,5 |  | 52 | 2,2 | 82,4 |  |
|  |  |  |  |  |  |  |  |  |
| **housing tenure** |  |  |  | p<0,001 |  |  |  | p<0,001 |
| rents | 1513 | 36,7 | 69,3 |  | 446 | 23,0 | 64,8 |  |
| owns with mortgage | 1119 | 25,8 | 85,3 |  | 243 | 11,5 | 80,2 |  |
| owns outright | 1594 | 37,5 | 75,7 |  | 1367 | 65,6 | 77,5 |  |
|  |  |  |  |  |  |  |  |  |
| **employment status** |  |  |  | p<0,001 |  |  |  | p<0,001 |
| inactive | 578 | 12,3 | 62,3 |  | 276 | 12,6 | 68,3 |  |
| employed | 2927 | 69,6 | 80,6 |  | 796 | 36,7 | 76,8 |  |
| unemployed | 329 | 7,6 | 63,2 |  | 94 | 4,4 | 60,8 |  |
| retired | 392 | 10,6 | 69,4 |  | 890 | 46,3 | 76,6 |  |
|  |  |  |  |  |  |  |  |  |
| **HEALTH BEHAVIOUR** |  |  |  |  |  |  |  |  |
| **smoking** |  |  |  | p<0,001 |  |  |  | p<0,001 |
| never smokers | 1713 | 40,6 | 76,3 |  | 1008 | 48,4 | 77,1 |  |
| current smokers | 1106 | 26,6 | 72,4 |  | 284 | 14,4 | 64,0 |  |
| ex-smokers | 940 | 21,8 | 83,4 |  | 374 | 18,2 | 80,0 |  |
| missing | 467 | 10,9 | 67,2 |  | 390 | 19,0 | 72,6 |  |
|  |  |  |  |  |  |  |  |  |

| ***(Table A1 continued)*** |  |  |  |  |  |  |  |  |
| --- | --- | --- | --- | --- | --- | --- | --- | --- |
| **HEALTHCARE ACCESS** |  |  |  |  |  |  |  |  |
| **complementary health insurance status** |  |  |  | p<0,001 |  |  |  | p<0,001 |
| none | 166 | 4,1 | 61,7 |  | 100 | 4,8 | 53,1 |  |
| private | 3670 | 88,9 | 78,1 |  | 1859 | 91,2 | 77,1 |  |
| free coverage for low income | 390 | 7,0 | 55,6 |  | 97 | 4,0 | 52,6 |  |
|  |  |  |  |  |  |  |  |  |
| **long-term illness fee exemption** |  |  |  | p<0,001 |  |  |  | p<0,001 |
| yes | 554 | 13,1 | 68,7 |  | 405 | 20,0 | 68,0 |  |
| no | 3672 | 86,9 | 76,9 |  | 1651 | 80,0 | 76,6 |  |
|  |  |  |  |  |  |  |  |  |
| **HEALTHCARE UTILIZATION** |  |  |  |  |  |  |  |  |
| **physicians consulted within the last 12 months** | |  |  | p<0,001 |  |  |  | p<0,001 |
| at least one gynecologist | 1694 | 40,1 | 95,3 |  | 558 | 26,4 | 92,2 |  |
| other physician(s) | 2241 | 53,2 | 64,5 |  | 1372 | 67,6 | 70,5 |  |
| none | 258 | 6,0 | 49,3 |  | 102 | 4,9 | 50,4 |  |
| missing | 33 | 0,7 | 44,7 |  | 24 | 1,2 | 41,7 |  |
|  |  |  |  |  |  |  |  |  |
| a N refers to number of women in unweighted sample | |  |  |  |  |  |  |  |
| b weighted percentages (to account for the survey’s sampling design and overall non-response) | | | | | | | | |
| c weighted screening rate (to account for the survey’s sampling design and overall non-response) | | | | | | | | |
| d chi test for distribution of screening rates in univariate analysis | | | |  |  |  |  |  |
